# Occurrence and Function of the Na^+^-Translocating NADH:Quinone Oxidoreductase in *Prevotella* spp.

**DOI:** 10.3390/microorganisms7050117

**Published:** 2019-04-27

**Authors:** Simon Deusch, Eva Bok, Lena Schleicher, Jana Seifert, Julia Steuber

**Affiliations:** 1 Institute of Animal Science, University of Hohenheim, 70599 Stuttgart, Germany; deusch@uni-hohenheim.de; 2 Institute of Microbiology, University of Hohenheim, 70599 Stuttgart, Germany; eva.bok@student.uni-tuebingen.de (E.B.); lena.schleicher@uni-hohenheim.de (L.S.)

**Keywords:** Prevotellaceae, NQR, *P. bryantii* B_1_4, proteomics

## Abstract

Strictly anaerobic *Prevotella* spp. are characterized by their vast metabolic potential. As members of the Prevotellaceae family, they represent the most abundant organisms in the rumen and are typically found in monogastrics such as pigs and humans. Within their largely anoxic habitats, these bacteria are considered to rely primarily on fermentation for energy conservation. A recent study of the rumen microbiome identified multiple subunits of the Na^+^-translocating NADH:quinone oxidoreductase (NQR) belonging to different *Prevotella* spp. Commonly, the NQR is associated with biochemical energy generation by respiration. The existence of this Na^+^ pump in *Prevotella* spp. may indicate an important role for electrochemical Na^+^ gradients in their anaerobic metabolism. However, detailed information about the potential activity of the NQR in *Prevotella* spp. is not available. Here, the presence of a functioning NQR in the strictly anaerobic model organism *P. bryantii* B_1_4 was verified by conducting mass spectrometric, biochemical, and kinetic experiments. Our findings propose that *P. bryantii* B_1_4 and other *Prevotella* spp. retrieved from the rumen operate a respiratory NQR together with a fumarate reductase which suggests that these ruminal bacteria utilize a sodium motive force generated during respiratory NADH:fumarate oxidoreduction.

## 1. Introduction

Gram-negative, obligate anaerobic bacteria of the Prevotellaceae family represent the prevailing and most numerous species within the rumen [1,2,3] and also commonly occur in the intestinal tract of humans and pigs [4,5,6]. Members of the Prevotellaceae family exhibit versatile metabolic capabilities such as the utilization of peptides, proteins, monosaccharides, and non-structural plant polysaccharides [7,8,9]. As a representative of the Prevotellaceae family, the type strain *Prevotella bryantii* B_1_4, isolated from the rumen [10], is likewise a typical inhabitant of digestive tract ecosystems. In addition to its ability to degrade proteins and peptides [11] this bacterium exhibits an extensive xylanolytic activity [10,12]. Despite the numerical and metabolic significance of *Prevotella* spp. in gastrointestinal environments, little is known about their metabolism and growth requirements. The oxidative branches of amino acid [13] and glucose [14] fermentation by *P. intermedia* and *P. nigrescens* have been studied, but the reductive pathways leading to the regeneration of NAD^+^ have not been investigated and the overall redox balance has not been taken into account. When *P. copri* is grown in defined minimal medium with glucose as sole carbon source, glycolysis represents the major fermentative pathway coupling the respiratory NADH:fumarate oxidoreduction with the redox cycling of ferredoxin [15].

The rumen constitutes a strictly anaerobic ecosystem characterized by pH values between 5.5 and 6.9, high buffering capacities, and low redox potentials of −400 to −350 mV which favor anaerobic metabolism. Within the rumen, fermentation respectively substrate-level phosphorylation is considered to be the principle strategy for energy conservation of bacteria and outweighs anaerobic respiration [16]. A comprehensive study of the rumen microbiome identified copious proteins of the Prevotellaceae family including various enzymes belonging to *P. bryantii* B_1_4 [17]. Several subunits of the respiratory Na^+^-translocating NADH:quinone oxidoreductase (NQR) were discovered among the respective proteins suggesting that metabolically active *Prevotella* spp. operate redox-driven Na^+^ pumps within their natural habitats as reported recently for *Prevotella copri* [15]. Thus, it may be assumed that electrochemical Na^+^ gradients potentially play an important role in the anaerobic metabolism of these ruminal bacteria.

The NQR is embedded in the inner membrane of several Gram-negative bacteria [18,19]. It is composed of the six subunits NqrABCDEF that include flavins and FeS clusters as redox cofactors [20]. Oxidation of NADH is catalyzed by subunit NqrF and is linked to the reduction of ubiquinone by subunit NqrA. This exergonic redox reaction drives the endergonic transport of Na^+^ through the membrane-bound subunit NqrB [21]. Thus, an electrochemical Na^+^ gradient is established which drives important cellular processes such as nutrient uptake, motility, or extrusion of toxic compounds [22]. The occurrence of the NQR complex in a strict anaerobe such as *P. bryantii* B_1_4, examined here, suggests that this Na^+^ pump constitutes a significant part of an anaerobic respiratory chain beneficial for survival in the sodium-rich rumen ecosystem.

Sequences encoding the six NQR subunits were identified in the genomes of several bacterial clades from distinct habitats with divergent metabolic requirements including certain species of the phylum of Bacteroidetes [18]. Comparative genomics detected sequences of the NQR subunits in 90 bacterial taxa that also included *Bacteroides fragilis* YCH46 and *Bacteroides* sp. 2_1_33B which belong to the family of Bacteroidaceae closely related to the family of Prevotellaceae [18]. Furthermore, a recent study found components similar to the NQR complex of *Vibrio alginolyticus* in seven strains of the genus *Bacteroides* [23]. Lately, the presence of genes encoding the NQR was noted for *Prevotella bryantii* B_1_4 and also the potential interplay of the NQR with a fumarate reductase was described [24]. In this study, we confirm the existence of an active NQR in *P. bryantii* B_1_4 by using mass spectrometric, biochemical, and kinetic approaches and discuss its potential involvement in anaerobic respiration and metabolism.

## 2. Materials and Methods 

### 2.1. Analysis of Prevotellaceae-Derived Proteins from the Rumen

A previously published study employed liquid chromatography–tandem mass spectrometry (LC–MS/MS) and the software MaxQuant (v. 1.5.3.8, Max Planck Institute of Biochemistry–Martinsried, Bavaria, Germany) to quantify bacterial proteins in the rumen ecosystem. The obtained metaproteomic dataset included a large number of proteins belonging to various members of the Prevotellacea family [17]. The subcellular localization of the respective proteins was inferred using the Cello algorithm [25] for Gram-negative bacteria which differentiates between cytoplasmic, periplasmic, inner and outer membrane proteins and also assigns extracellular proteins. The protein sequences were screened for hydrophobic stretches to identify membrane-affiliated motifs using the TMHMM (transmembrane alpha-helices Hidden Markov Model) Server v. 2.0 algorithms [26], to assign α-helices and the PRED-TMBB2 (prediction of transmembrane beta-barrels) algorithms [27], to find β-barrel outer membrane proteins. Furthermore, the Prevotellaceae-derived protein dataset was searched manually for subunits of the Na^+^-translocating NADH:quinone reductase (NQR) and other respiratory enzymes also including the related ferredoxin:NAD^+^ oxidoreductase complex (RNF) complex.

### 2.2. Organism and Growth Conditions

*P. bryantii* B_1_4 [10] was kindly provided by Nest McKain (Rowett Research Institute, Aberdeen, UK) and cultivated in the liquid form of Hobson´s M2 medium [28] under an O_2_-free CO_2_ atmosphere at 39 °C as demonstrated before [29]. Despite the availability of defined media without rumen fluid [15,30], Hobson’s M2 medium [28] was used to simulate conditions as close as possible to the rumen environment. Cultivation was conducted in Hungate tubes and in serum vials for larger volumes. Inoculum volume was 5% (*v*/*v*) of an active culture or glycerol stock. To achieve a consistent media composition, rumen fluid of a fistulated Holstein–Frisian cow (approved by Regierungspräsidium Stuttgart; V263/09 TE) was sampled once, 1 h prior to the morning feeding using a vacuum pump. The animal was fed twice a day with 4.5 kg hay diet containing 12% concentrate (20% soybean, 17% corn, 25% barley, 28% wheat, 4% molasses, 6% minerals). 

### 2.3. Proteome Extraction, Fractionation and Sodium Dodecyl Sulfate PolyAcrylamide Gel Electrophoresis (SDS-PAGE)

Exponentially growing cells (wet weight 0.5 g) were harvested at 15,000× *g* for 15 min at 4 °C, washed in 5 mL of 50 mM Tris-HCl (pH 7.5), and resuspended in 4 mL of 50 mM Tris-HCl (pH 7.5; 10 mM MgCl_2_; 1 mM phenylmethylsulfonyl fluoride) followed by sonication on ice using a tip sonicator (Ultrasonic Processor UP50H, Hielscher, Germany) for four times 3 min (amplitude 50%; cycle 0.5). The cell debris fraction (CD) was removed by centrifugation at 15,000× *g* for 15 min at 4 °C and stored at −20 °C until further processing. The membrane fraction (MP) was enriched from the crude extract by centrifugation at 90,000× *g* for 1 h at 4 °C. The supernatant containing soluble proteins was discarded and the MP fraction was washed once in 1 mL 50 mM Tris-HCl (pH 7.5) and collected by centrifugation at 90,000× *g* for 1 h at 4 °C. Please note that this single washing step did not result in complete removal of cytoplasmic and periplasmic proteins from the enriched membranes. Pellets of the MP and CD fractions were resolved in 50 µL of 20 mM Tris-HCl (pH 7.5), and 100 µL of 20 mM Tris-HCl (pH 7.5; 2% (*w*/*v*) sodium dodecyl sulfate) were added. The samples were incubated for 5 min at 60 °C and 1200 rpm, and 1 mL of 20 mM Tris-HCl (pH 7.5; 0.1 mg/mL MgCl_2_; 1 mM phenylmethylsulfonyl fluoride; 1 μL/mL Benzonase–Novagen) was added followed by sonication on ice with a tip sonicator (Ultrasonic Processor UP50H, Hielscher, Germany) for two times 1 min (amplitude 50%; cycle 0.5). Subsequently, nuclease digestion was performed for 10 min at 37 °C and 1400 rpm. The supernatants of the MP and CD fractions were collected after centrifugation at 10,000× *g* for 10 min at 4 °C and stored at −20 °C. The amount of protein was quantified by the bicinchoninic acid assay [31] using bovine serum albumin as standard.

Aliquots of 60 µg of each fraction were precipitated by incubation in 1 mL 20% precooled trichloroacetic acid for 30 min at 4 °C and centrifugation at 12,000× *g* for 15 min at 4 °C. The protein pellets were washed twice in precooled acetone, dried by vacuum centrifugation and resuspended in 30 μL Laemmli buffer [32]. After incubation for 5 min at 95 °C to reduce disulfide bonds, 15 µL were applied on a one-dimensional sodium dodecyl sulfate polyacrylamide gel electrophoresis (SDS-PAGE) that was carried out as reported previously [33] using 10% acrylamide/bisacrylamide (37.5:1.0) gels. Electrophoresis was started at 20 mA until proteins entered the separation gel and was completed at 40 mA. The gel was scanned by a CanoScan LIDE100 (Canon, Tokyo, Japan) and lanes of interest were cut out for proper illustration using IrfanView (v. 4.51, Irfan Škiljan, Vienna, Austria). Each Coomassie-stained gel lane was cut into pieces and stored at −20 °C until subsequent trypsin digestion. 

### 2.4. Isolation of Membranes, Solubilization and Blue Native Polyacrylamide Gel Electrophoresis (BN-PAGE)

Exponentially growing cells were harvested at 15,000× *g* for 30 min at 4 °C and washed twice in 20 mM Tris-H_2_SO_4_ (pH 7.5). Cell disruption was performed as described earlier [34], with some modifications. The procedure was kept chloride-free to prevent Ag^+^ precipitation during kinetic experiments. A wet weight of 10 g of cells was resuspended in 30 mL 20 mM Tris-H_2_SO_4_ (pH 7.5; 50 mM Na_2_SO_4_; 5 mM MgSO_4_; 1 mM dithiothreitol; 1 mM PMSF; 0.1 mM diisopropyl fluorophosphate; traces of DNase I – Roche, Basel, Switzerland). The suspensions were passed three times through an EmulsiFlex high pressure homogenizer at 20,000 psi and 4 °C. Cell debris and unbroken cells were removed by centrifugation at 27,000× *g* for 30 min at 4 °C. Membranes were collected by ultracentrifugation at 257,000× g for 1 h at 4 °C and washed in 20 mM Tris-H_2_SO_4_ (pH 7.5; 50 mM Na_2_SO_4_, 5% (*v*/*v*) glycerol). Suspensions of isolated membranes were stored in liquid nitrogen.

The blue native polyacrylamide gel electrophoresis (BN-PAGE) was carried out as noted formerly [35], with minor modifications. To solubilize the membranes, 10 mg of membrane proteins were gently shaken over night at 4 °C in 1 ml 20 mM Tris-H_2_SO_4_ (pH 7.5) with 1% or 2% (*w*/*v*) Triton X-100 followed by centrifugation at 156,425× *g* for 1 h and 4 °C. Solubilized and non-solubilized membranes were diluted in 50 mM aminocaproic acid with 0.3% (*w*/*v*) Coomassie Brilliant Blue G250 and 200 µg were subjected to a polyacrylamide gradient gel (5–20%). The gel was run at 100 V until the frontline passed one third of the gel. To complete the run at 200 V, the cathode buffer (pH 7; 50 mM Tricine; 15 mM BisTris; 0.02% Coomassie Brilliant Blue G250) was replaced by a Coomassie-free buffer (pH 7; 50 mM Tricine; 15 mM BisTris). The BN-PAGE was stained with Coomassie Brilliant Blue G250 and scanned by a CanoScan LIDE100 (Canon, Tokyo, Japan). Visible gel bands of membranes solubilized with 1% or 2% (*w*/*v*) Triton X-100 were excised and stored at −20 °C until subsequent trypsin digestion.

### 2.5. Trypsin Digestion, Liquid Chromatography–Tandem Mass Spectrometry (LC–MS/MS) Measurements and Bioinformatic Processing

The parts of the excised gel lanes from the SDS-PAGE (2.3) and the single gel bands of solubilized membranes from the BN-PAGE (2.4) were subjected to in-gel trypsin (Promega, Madison, WI, USA) digestion overnight [36]. The obtained tryptic peptides were purified and desalted using C_18_ microcolumns (Merck Millipore, Burlington, MA, USA) and dried by vacuum centrifugation.

Peptide samples were reconstituted in 20 µL 0.1% formic acid and analyzed using an ACQUITY nano-UPLC (ultra-performance liquid chromatography) system (Waters, Milford, MA, USA) coupled to a LTQ-Orbitrap XL hybrid mass spectrometer (Thermo Fisher Scientific, Waltham, MA, USA). Of each sample, 5 µL were concentrated and desalted on a C_18_ precolumn (180 µm × 2 cm, 5 µm) and separated on a BEH 130 C_18_ reversed phase column (75 µm × 25 cm, 1.7 µm). Chromatography was performed from 1% to 40% acetonitrile in 0.1% formic acid with a gradient time of 240 min. The LTQ-Orbitrap XL was operated in the positive ion mode under control of the XCalibur 2.1 software (Thermo Fisher Scientific, Germany). Survey spectra were detected in the mass range of 250–1800 *m*/*z* in the Orbitrap mass analyzer with a resolution of *r* = 60,000 at 400 *m*/*z*. Data dependent tandem mass spectra were generated for the seven most abundant peptide precursors in the linear ion trap. For all measurements using the Orbitrap detector, internal calibration was performed using lock-mass ions from ambient air.

Raw mass spectrometry (MS) and MS/MS data were processed by Thermo Proteome Discoverer software (v. 1.4.1.14, Thermo Fisher Scientific, Waltham, MA, USA) and the Mascot engine (v. 2.4, Matrix Science, London, UK) by searching independently against the UniProtKB/TrEMBL database (v. 12/2017, European Bioinformatics Institute, Hinxton Cambridge, UK) for *Prevotella bryantii* (3779 sequences). Oxidation of methionine was set as variable modification and carbamidomethylation of cysteine as fixed modification. Precursor ion tolerance was defined at 10 ppm and fragment ion tolerance to 0.02 Da with a maximum of one missed trypsin cleavage. Furthermore, the Spectrum Grouper node was employed with a precursor mass tolerance of 10 ppm and a max. retention time difference of 90 s, and the Percolator node was activated with a false discovery rate of 1%. The default filter was set to a minimum of two peptides per protein and a Mascot Significance threshold of 0.05 including a peptide, protein and PSM false discovery rate below 1%. Protein grouping was enabled with a minimum PSM confidence of medium and a delta Cn better than 0.15, strict maximum parsimony principle was applied.

The subcellular localization and membrane-associated motifs (α-helices and β-barrels) of the identified proteins were assigned as described for the metaproteomic dataset (2.1) and the proteins were screened again for subunits of the NQR complex, the RNF complex and other respiratory enzymes including the electrogenic NADH:quinone oxidoreductase (NDH-I). Multiple sequence alignments were conducted using the Clustal Omega algorithm of the European Bioinformatics Institute with default settings (ebi.ac.uk/Tools/msa/clustalo/). In silico trypsin digestion to find shared tryptic peptides was performed using the ExPASy PeptideCutter of the Swiss Institute of Bioinformatics (web.expasy.org/peptide_cutter/).

### 2.6. Detection of NADH:Quinone Oxidoreductase (NQR) Homologues by In-Gel Fluorescence

The presence of NQR homologues was determined in detecting flavin mononucleotides (FMNs) covalently bound to the NqrB and NqrC subunits by in-gel fluorescence as demonstrated before [37], with slight modifications. Protein concentration was determined by the bicinchoninic acid assay [31] and 150 µg of all subcellular fractions obtained during the isolation of membranes (as conducted in 2.4) were subjected to SDS-PAGE [33]. In-gel fluorescence of proteins with covalently bound FMN chromophores was detected immediately after electrophoresis using the ImageQuant LAS 4000 imager (λ_excitation_ = 460 nm, emission filter = Y515 Cy™2). Fluorescent proteins with molecular masses of 25, 50 and 75 kDa served as molecular size markers (Precision Plus Protein™ WesternC™ Standards, BioRad; excitation, red LED; emission filter, R670 Cy5). The contrast of the gel picture was slightly increased using IrfanView (v. 4.51) to achieve a more appropriate visualization of the fluorescent bands.

### 2.7. Inhibition of the NADH:Quinone Oxidoreduction Activity by Ag^+^

Inhibition of NADH oxidation was measured in an assay buffer (20 mM Tris-H_2_SO_4_; 50 mM Na_2_SO_4_; 5% (*v*/*v*) glycerol; 100 µM ubiquinone-1; 150 µM NADH) at 25 °C by a Diode-Array Spectrophotometer (HP 8452A) using 150 µg of membranes (obtained as described in 2.4). In advance, membranes were diluted in 20 mM Tris- H_2_SO_4_ (5% (*v*/*v*) glycerol) and preincubated with varying amounts of AgNO_3_ (0–2.5 µM) for 5 min at 4 °C. The reaction was started by adding the preincubated membranes to the assay buffer. Subsequently, NADH oxidation was monitored by following the absorbance at 340 nm. Specific activities were determined based on the molar extinction coefficient of NADH at 340 nm (ε_340_ = 6.22 mM^−1^ cm^−1^) applying Beers’ law.

### 2.8. Stimulation of the NADH:Quinone Oxidoreduction Activity by Na^+^

The enzyme assays for sodium stimulation of the NADH oxidation by membranes of *P. bryantii* B_1_4 were performed at 25 °C in 20 mM Tris-HCl (pH 7.5) with increasing concentrations (0 to 500 µM) of NaCl or KCl, 100 µM NADH dipotassium salt, 100 µM ubiquinone-1, and 50 µg of isolated membranes (obtained as described in 2.4). The initial rates were recorded on a SPECORD^®^ S600 UV VIS spectrophotometer (Analytik Jena, Jena, Germany) and NADH oxidation was followed at 340 nm (ε_340_ = 6.22 mM^−1^ cm^−1^). The residual Na^+^ concentration in each sample was 40 µM. Sodium concentrations were determined with an Agilent Technologies 200 Series AA spectrometer.

## 3. Results and Discussion

### 3.1. Identification of Na^+^-Translocating NADH:Quinone Oxidoreductase Subunits by LC–MS/MS

#### 3.1.1. Ruminal Prevotellaceae Proteins Include Subunits of the NQR

**Figure 1:**
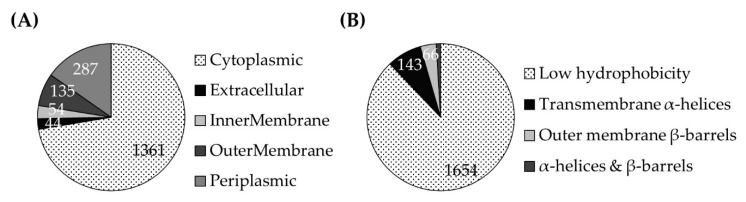
Sequence-based prediction of subcellular localization and hydrophobicity of 1,881 proteins of the Prevotellaceae family as identified in the rumen ecosystem. (**A**) Results obtained from the Cello algorithm (cello.life.nctu.edu.tw/) which differentiates between four cellular compartments and extracellular proteins. (**B**) The combined results of the TMHMM Server v. 2.0 (cbs.dtu.dk/services/TMHMM/) and the PRED-TMBB2 (compgen.org/tools/PRED-TMBB2/) algorithms which assign hydrophobic stretches (α-helices and β-barrels).

A comprehensive study investigated the rumen microbiome and quantified a total of 8163 bacterial proteins in three distinct rumen fractions of three individual cows that were fed rotationally with varying diets [17] which revealed that a major part of 1881 proteins belonged to the family of Prevotellaceae of which 49 proteins were assigned to *P. bryantii* B_1_4 (Table S1). About 72% of the Prevotellaceae-derived proteins were predicted to be cytoplasmic proteins, above 15% belonged to the periplasmic space, and 10% were assigned to inner and outer membrane proteins (Figure 1A and Table S1) which emphasized the capability of mass spectrometry-based analyses to identify subunits of membrane-associated proteins [38]. A total of above 12% of the respective protein sequences were predicted to contain hydrophobic stretches that were assigned to membrane-bound α-helices and β-barrels (Figure 1B and Table S1).

The 143 proteins exhibiting α-helices (Figure 1B and Table S1) included, inter alia, the NQR subunits NqrB, NqrC, and NqrF of *P. ruminicola*, and the NqrF of *Prevotella* sp. P5-119 (Table 1). Furthermore, four NqrA subunits of *P. ruminicola*, *P. timonensis*, *Prevotella* sp. CAG:1092, and *Prevotella* sp. S7 MS 2 were identified among the 1881 Prevotellaceae proteins. In addition, 15 other respiratory enzymes of *Prevotella* spp. such as the anaerobic electron transport complex protein RnfG, succinate dehydrogenases, or the fumarate reductase iron-sulfur protein of *P. bryantii* B_1_4 were found to be present (Table 1).

The NQR and the closely related ferredoxin:NAD^+^ oxidoreductase complex (RNF), which have a common ancestor, are redox-driven Na^+^-pumps [39]. Under anaerobic conditions, the RNF may also be employed in reverse electron transfer reactions for reduction of NAD^+^ yielding NADH [40,41]. Genes encoding the NQR were predicted for a variety of genomes from aerobic, microaerophilic, and even obligate anaerobic microorganisms such as *Bacteroides* spp. [18,23]. Generally, the NQR is the dedicated Na^+^ pump in aerobic or facultative anaerobic bacteria [42,43]. The occurrence of the Na^+^-translocating NQR in members of the Gram-negative Prevotellaceae family, which are obligate anaerobes, indicates that the generated sodium-motive force is a so far overlooked, but potentially important, factor for the survival of these species within their natural habitats.

To exclude false-positive protein identifications due to the large search databases that were employed for the bioinformatic processing of the rumen metaproteomic data and, thus, to confirm the presence of a Na^+^-translocating NQR in *Prevotella* spp., proteome fractions of the model organism *P. bryantii* B_1_4 [9] were analyzed by mass-spectrometry as follows.

#### 3.1.2. NQR Subunits Detected in Fractions of the *Prevotella bryantii* B_1_4 Proteome

**Figure 2:**
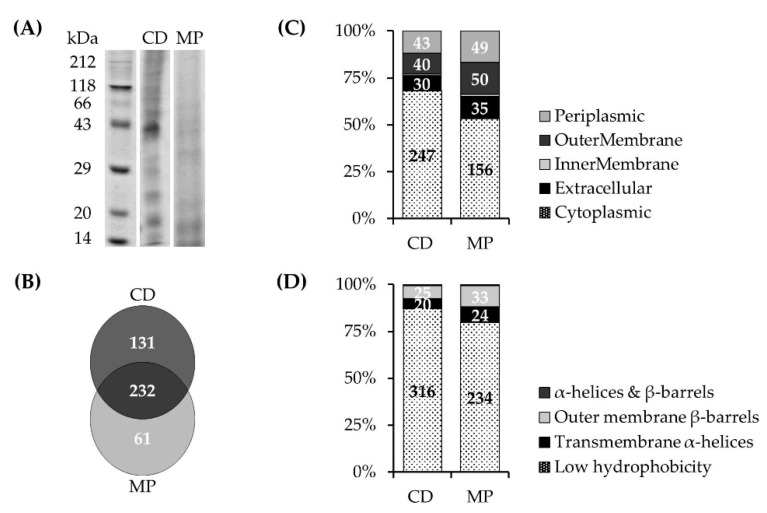
(**A**) Separation of proteins from subcellular fractions (MP, membrane proteins; CD, cell debris) of the *P. bryantii* B_1_4 proteome by sodium dodecyl sulfate polyacrylamide gel electrophoresis (SDS-PAGE). (**B**) Overlap of proteins identified in the MP fraction (293 proteins) and CD fraction (363 proteins). (**C**) Predicted subcellular localization of proteins (Cello). Cytoplasmic proteins detected in the MP fraction represent contaminations which were not completely removed during preparation of the MP fraction. (**D**) Membrane-affiliated motifs: α-helices and β-barrels of proteins identified in the MP and CD fractions (TMHMM Server v. 2.0 and PRED-TMBB2).

LC–MS/MS-based analyses of membrane protein (MP) and cell debris (CD) fractions obtained from *P. bryantii* B_1_4 cultures (Figure 2A) identified a total of 293 and 363 proteins respectively (Table S2). A total of 232 proteins were detected in both proteome fractions (Figure 2B) which indicated an insufficient separation of the subcellular fractions. More than 68% of proteins found in the CD fraction were predicted to be cytoplasmic proteins, about 12% belonged to the periplasmic space, and 11% were assigned to outer membrane proteins (Figure 2C). In contrast, 17% of the proteins of the MP fraction were outer membrane proteins and 17% belonged to the periplasm whereas 53% were allocated to the cytoplasm (Figure 2C). This indicates that soluble proteins were not completely removed from the membranes which were resuspended only once in Tris buffer. Inspection for hydrophobic stretches revealed that more than 20% of protein sequences of the MP fraction contained membrane-affiliated motifs while less than 13% of the CD proteins exhibited sequence sections assigned to α-helices and β-barrels (Figure 2D).

Searching for subunits of the NQR and other respiratory enzymes in the proteome datasets of *P. bryantii* B_1_4 confirmed the presence of the NqrA and NqrC in both, the CD and MP fractions (Table 2). Furthermore, the electron transport complex protein RnfG and other respiratory enzymes like succinate dehydrogenases, fumarate reductases, and subunits of the bacterial homologue to the eukaryotic complex I [44] were identified (Table 2). Note that *P. copri* [15] and members of the Bacteroidetes phylum [45] contain the so-called headless NDH-I complex. This membrane-bound protein complex contains 11 of the 14 subunits of the bacterial complex I (NDH-I). Since it lacks 3 peripheral subunits required for NADH oxidation, it will not contribute to the overall NADH oxidation activity of membranes from *Prevotella* spp.

To prevent the possibility of erroneously identifying subunits of the homologous RNF complex or the electrogenic NDH-I, multiple sequence alignments were carried out to determine the amino acid sequence identity of the NQR subunits and the RNF complex or the most similar subunits of the NDH-I complex. The highest sequence identity of 44.5% was obtained for the NqrE and the RnfA subunits whereas the residual alignments exhibited a negligible sequence identity (Table 3). Moreover, in silico trypsin digestion revealed that there are no shared tryptic peptides for all subunits of the NQR, RNF, and the headless NDH-I complex which ensures confident identification by LC–MS/MS and subsequent bioinformatic processing as further demonstrated by the number of unique peptides that were used for identification (Table 2).

However, in the course of the mass spectrometry-based analysis of fractions of the *P. bryantii* B_1_4 proteome, exclusively the NqrA and NqrC subunits were identified and an adequate separation of the membrane proteins was not achieved. Thus, to confirm the occurrence of a NQR complex and to identify the remaining NQR subunits in metabolically active *P. bryantii* B_1_4 cells, further validation was required and conducted as follows.

#### 3.1.3. NQR Subunits Identified in Solubilized Membranes of *Prevotella bryantii* B_1_4

Isolated membranes of *P. bryantii* B_1_4 were solubilized with Triton X-100 and subjected to a BN-PAGE (Figure S1) to be analyzed by LC–MS/MS. Mass spectrometric analyses of excised protein bands (Figure S1) identified 163, 186 and 212 proteins in bands A, B and C of the 1% Triton X-100 treatment respectively (Table S3). Regarding the membrane proteins solubilized in 2% Triton X-100, 139, 177 and 197 proteins were identified in the bands A, B and C respectively (Table S3). Subunits of the NQR, RNF, the headless NDH-I complex (Nuo), and other respiratory enzymes such as succinate dehydrogenases or fumarate reductases that were detected are listed in Table 4.

The bands B and C from solubilized membranes (of both, 1% and 2% Triton X-100 solubilisates) contained the NADH-oxidizing NqrF subunit (Table 4) whereas the NqrF was not detected in the respective bands A. Besides the more hydrophobic NqrE, all subunits of the NQR complex (NqrABCDF) were identified based on unique peptides in band B of the 1% Triton X-100 and band C of the 2% Triton X-100 treatment (Table 4). In contrast, the bands A of both Triton X-100 solubilisates exclusively contained the more hydrophilic NqrA subunit. 

The subunits NuoB, NuoCD, NuoH, NuoI, NuoL and NuoN of the headless NDH-I complex were also detected but with less confident identification scores, less unique peptides, and lower sequence coverages when compared to the identification parameters of the NQR subunits (Table 4). A recent study confirmed the expression of the headless NDH I complex I in *P. copri* by qRT-PCR [15]. As predicted from the genome, we did not detect the peripheral subunits NuoEFG (N-module) that are responsible for NADH oxidation in *P. bryantii* B_1_4 which further emphasizes the prevalent role of the NQR regarding NADH oxidation. Of the RNF complex, exclusively the electron transport protein RnfG was detected in the bands A and C of the 1% Triton X-100 and band C of the 2% Triton X-100 solubilisates, again with less certain identification parameters when compared to the NQR subunits.

### 3.2. Prevotella bryantii B_1_4 Contains a Functional NQR

#### 3.2.1. Detection of the Membrane-Bound Subunits NqrB and NqrC

The NQR complex comprises several flavin cofactors such as the flavin adenine dinucleotide (FAD) that is bound noncovalently to the NqrF subunit, riboflavin which is associated noncovalently with the NqrB subunit, and two flavin mononucleotides (FMNs) that are covalently attached to the NqrB and NqrC subunits by a phosphodiester bond at the threonine residues 236 and 225, respectively [46,47].

**Figure 3:**
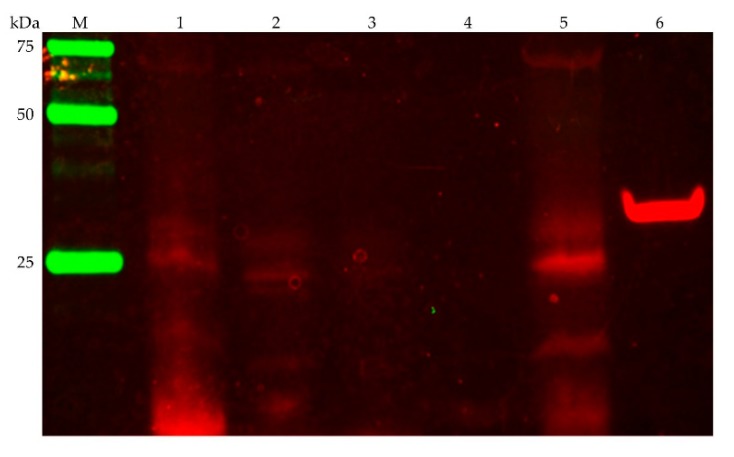
Detection of the membrane-bound NQR subunits NqrB and NqrC by in-gel fluorography. Cell fractions were separated by SDS-PAGE (0.15 mg protein per lane). (**M**) protein marker; (**1**) unbroken cells; (**2**) crude extract; (**3**) soluble fraction; (**4**) cleared supernatant of washed membranes after centrifugation; (**5**) washed membranes; (**6**) positive control: truncated NqrC_33-257_ of the *Vibrio cholerae* NQR complex comprising the covalently attached FMN but lacking the N-terminal transmembrane helix.

The presence of the NqrB and NqrC subunits of *P. bryantii* B_1_4 was verified by in-gel fluorescence detection of covalently bound FMN chromophores (Figure 3). This is shown best in lane no. 5 of Figure 3 that comprises the washed membranes which exhibit a fluorescent band at about 25 kDa. It was reported before that the NqrB and NqrC subunits migrate together under these conditions and appear as a single band at about 25 kDa [48]. The NqrB and NqrC subunits of *P. bryantii* B_1_4 exhibit a length of 385 and 210 amino acids with a mass of 41.7 and 23.2 kDa, respectively. As a positive control, a truncated NqrC_33–257_ of *Vibrio cholerae* was used (Figure 3, lane 6) which lacks the N-terminal transmembrane helix. Please note that hydrophobic proteins like the NqrB and NqrC subunits migrate faster in SDS-PAGE [47].

In accordance with the observed fluorescence of proteins that run at a comparable weight in the SDS-PAGE and which we assigned as NqrB and NqrC, the threonine residues with covalently attached FMN cofactors in NqrB and NqrC of the *V. cholerae* NQR complex [47] are as well conserved in the corresponding NQR subunits of *P. bryantii* B_1_4.

#### 3.2.2. NADH Dehydrogenase Activity Is Inhibited by Micromolar Ag^+^

The preceding findings confirmed the presence of a NQR complex in membranes of growing *P. bryantii* B_1_4 cells but still did not provide evidence to answer the question if the NQR complex is truly functional and active. Therefore, we studied the inhibition of NADH oxidation by *P. bryantii* B_1_4 membranes using micromolar Ag^+^ concentrations. Silver ions particularly inhibit NADH oxidation of the NQR but do not affect the NDH-I complex [49].

**Figure 4:**
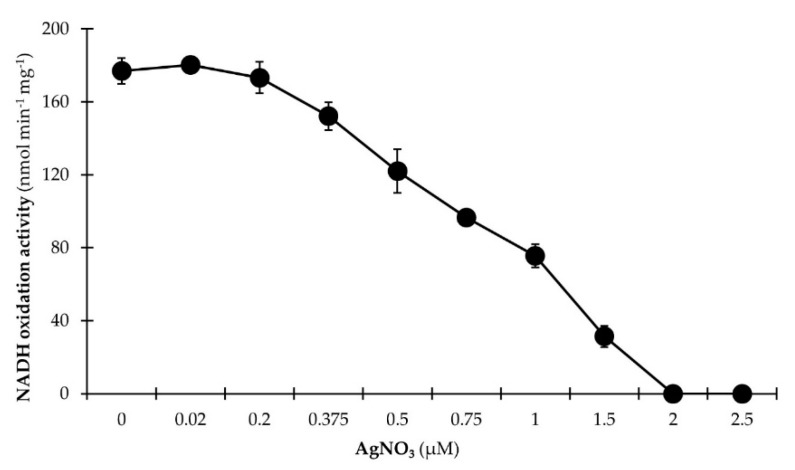
Ag^+^ inhibits NADH:quinone oxidoreduction activity of *P. bryantii* B_1_4 membranes. Membranes prepared in chloride-free buffers (aliquots of 150 µg protein) were incubated with varying amounts of AgNO_3_ (0–2.5 µM) for 5 min at 4 °C. Membrane aliquots were added to the reaction buffer (20 mM Tris H_2_SO_4_, 5 % glycerol, 50 µM Na_2_SO_4_, 150 µM NADH, 100 µM ubiquinone-1), and the oxidation of NADH was followed spectrophotometrically at 25 °C. Mean values and SD are from *n* = 3 measurements.

Membranes of the wild type *Vibrio cholerae* were reported to have a NADH dehydrogenase activity of about 0.4–0.5 μmol min^−1^ mg^−1^ [50] whereas membranes of *P. bryantii* B_1_4 exhibited an activity of about 0.2 μmol min^−1^ mg^−1^. The NQR is specifically inhibited by micromolar concentrations of Ag^+^ whereas the activity of other respiratory NADH dehydrogenases such as the electrogenic NDH-I (Nuo) or the non-electrogenic NADH dehydrogenase (NDH-II) is not affected [51]. As depicted in Figure 4, the half-maximum inhibition of NADH:quinone oxidoreduction activity of native membranes from *P. bryantii* B_1_4 was already achieved at a concentration of about 0.75 µM AgNO_3_ indicating that oxidation of NADH by membranes of *P. bryantii* B_1_4 is catalyzed predominantly by the NQR.

#### 3.2.3. NADH Dehydrogenase Activity Is Stimulated by Na^+^

**Figure 5:**
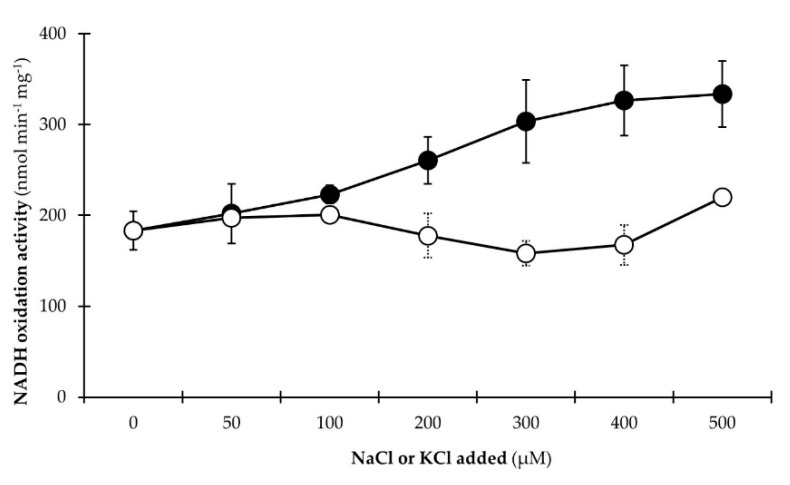
Stimulation of NADH:quinone oxidoreduction activity of *P. bryantii* B_1_4 membranes by Na^+^. The assay mixture contained membranes (aliquots of 50 µg protein), 20 mM Tris-HCl (pH 7.5), 100 µM NADH, and 100 µM ubiquinone-1. To the assay mixture, 0–500 µM NaCl (●) or KCl (○) were added. The residual Na^+^ concentration in every samples was 40 µM. The oxidation of NADH was followed spectrophotometrically at 25 °C. Mean values and SD are from *n* = 3 measurements.

To further confirm the notion of an active NQR complex and to verify the results of the previous experiment that used micromolar concentrations of Ag^+^ to inhibit NADH oxidation activity, the following kinetic experiment analyzed the stimulating effects of increasing sodium concentrations on the NADH oxidation activity of isolated membranes from *P. bryantii* B_1_4 (Figure 5). In parallel, identical amounts of KCl were used as a control for the Na^+^-dependent stimulation of NADH oxidation activity. 

Increasing amounts of sodium caused an enhanced NADH oxidation activity of *P. bryantii* B_1_4 membranes whereas the control using KCl instead of NaCl did not affect the NADH dehydrogenase activity. NADH oxidation activity of *P. bryantii* B_1_4 membranes increased from 223 nmol min^−1^ mg^−1^ at a NaCl concentration of 100 µM to a maximum of 334 nmol min^−1^ mg^−1^ at a NaCl concentration of 500 µM. Please note that the residual Na^+^ concentration in assay buffers (40 µM) would be sufficient for half-maximal activity of purified NQR from *Vibrio cholera* [52], which explains the basal activity of *P. bryantii* B_1_4 membranes without added salts (Figure 5). The inhibition of NADH oxidation activity by silver ions [49,53], and the stimulation of NADH oxidation by Na^+^ [54] are hallmark features for the presence of NQR in bacterial membranes, and demonstrate that *P. bryantii* B_1_4 membranes also contain active NQR. In contrast, the control experiment revealed a NADH oxidation activity of 201 nmol min^−1^ mg^−1^ at a KCl concentration of 100 µM and 220 nmol min^−1^ mg^−1^ at a KCl concentration of 500 µM which emphasized the prevalent role of the NQR complex in respiratory NADH oxidation by *P. bryantii* B_1_4.

## 4. Conclusions

**Figure 6:**
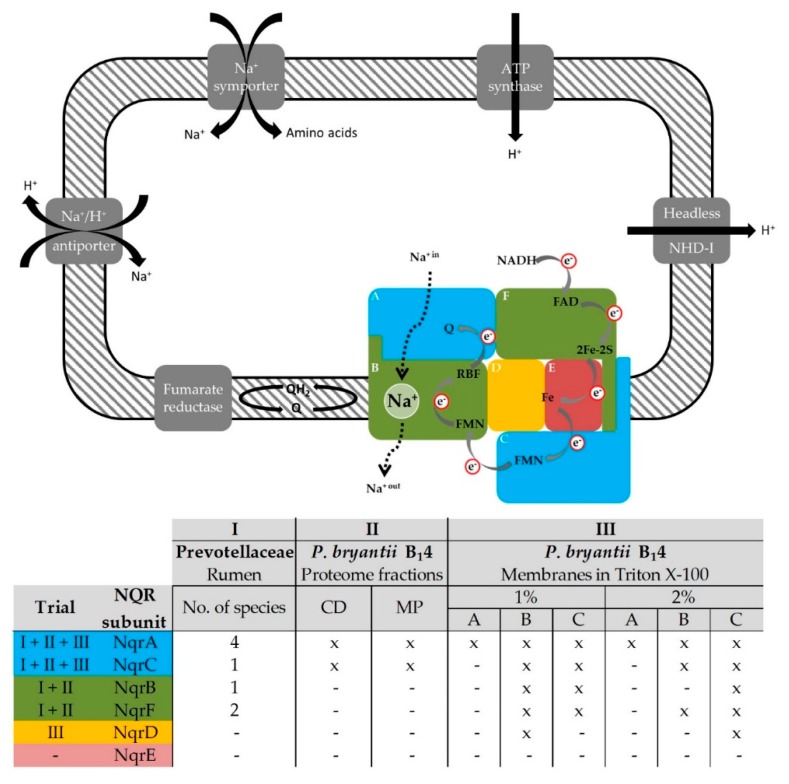
Critical role of the NQR in energy conservation of members of the Prevotellaceae family. Upper panel: The NQR consists of the six subunits NqrABCDEF and catalyzes the oxidation of NADH and reduction of quinone (Q) under translocation of Na^+^ (broken arrow). This overall reaction requires FAD and a 2Fe-2S protein in subunit NqrF, an iron (Fe) site in subunits NqrDE, one FMN in each subunit NqrC and NqrB, a riboflavin and a presumed Na^+^ binding site in NqrB, and a quinone (Q) binding site in NqrA. Electron (e^−^) transfer steps are indicated by grey arrows. Fumarate may constitute the final electron acceptor receiving electrons from quinol. The sodium motive force generated by the NQR may be used for sodium-symporters or the establishment of a proton gradient via Na^+^/H^+^ antiporters or the headless NDH-I complex to drive ATP regeneration. Lower panel: Nqr subunits identified in the rumen or in *P. bryantii* B_1_4 by mass spectrometry in three experimental trials (I, II, III) indicated by color code.

The identification of NQR subunits from five different *Prevotella* spp. in the metaproteomic dataset obtained from the rumen [17] and the fact that this respiratory complex pumps Na^+^ exclusively, suggested an important role of electrochemical Na^+^ gradients in the anaerobic energy metabolism and physiology of these major inhabitants of the rumen ecosystem. For the first time, to the best of our knowledge, the presence of a functioning NQR complex in the strictly anaerobic *P. bryantii* B_1_4 was verified by mass spectrometric, biochemical and kinetic experiments. Subunits of the NQR that were identified in the rumen and in *P. bryantii* B_1_4 are summarized in Figure 6. The unidentified NqrE subunit comprises only two tryptic peptides at single charge state and three peptides at double charge state that match the precursor mass filter of 300 to 1800 *m*/*z* of the LTQ-Orbitrap XL mass spectrometer. For comparison, the NqrD subunit exhibited a total of 7 tryptic peptides of detectable length (at each charge state). Thus, the low number of detectable peptides derived from NqrE prevented their identification.

The H^+^-translocating NDH-I complex (Nuo) is present in many bacterial species [55,56] but, according to genome predications, *P. bryantii* B_1_4 contains the headless NDH-I complex that lacks the subunits NuoEFG (N-module) which are responsible for the oxidation of NADH. The 11-subunit NDH-I complex was found, amongst others, in Bacteroidetes species [45] and the presence of the headless NDH-I complex I in *P. copri* was confirmed by quantitative reverse transcription polymerase chain reaction (qRT-PCR) [15]. The current mass spectrometric experiments did not identify the NuoEFG subunits in *P. bryantii* B_1_4 which further emphasized the dominant role of the NQR in oxidation of NADH.

The subunits NuoL, NuoM and NuoN of the NDH-I complex are homologues to the Na^+^/H^+^ gradient antiporter Mrp [57] which is capable of converting sodium gradients to proton gradients. Thus, it is possible that the headless NDH-I complex performs in a similar way. *P. bryantii* B_1_4 contains a proton gradient-driven ATP synthase and the sodium motive force generated by the NQR might be utilized to build up a proton gradient via Na^+^/H^+^ antiporters and possibly also by the headless NDH-I complex in order to regenerate ATP (Figure 6). Further studies are required to clarify the possible role of this complex in respiration or secondary cation/H^+^ antiport.

The electrochemical Na^+^ gradient generated by the NQR could also be used in sodium-symporters (Figure 6) as reported for the ruminal *Streptococcus bovis* that utilizes the sodium motive force for the import of amino acids [58]. Sodium dependent uptake of amino acids was reported for several ruminal bacteria [59].

*Prevotella* spp. represent the predominant bacterial genus in the rumen and are involved in the maintenance of the redox potential and influence the metabolite profile. Within their natural habitat, *Prevotella* spp. were considered to rely exclusively on fermentation for energy conservation. Our findings that *Prevotella* spp. retrieved from the rumen and *P. bryantii* B_1_4 operate a respiratory Na^+^-translocating NADH:quinone oxidoreductase which is probably linked to a fumarate reductase suggest that these ruminal bacteria critically depend on a sodium motive force generated during respiratory NADH:fumarate oxidoreduction. The NQR is robust voltage-generating machinery [37] and additional ATP yields from anaerobic respiration and the interlinked recovery of NAD^+^ represents a highly beneficial feature for survival and competition in the rumen ecosystem.

Regarding *Prevotella* spp. and *P. bryantii* B_1_4 we propose that reduced NADH produced by fermentation is oxidized by the NQR using fumarate as final electron acceptor (Figure 6) since subunits of the fumarate reductase including iron-sulfur proteins and flavoproteins were also detected in the mass spectrometric experiments (Tables S1–S3). The observation of a fumarate reductase in membranes from *P. bryantii* B_1_4 and the presence of the NQR suggests that NADH:fumarate oxidoreduction under generation of a sodium motive force (Figure 6) is an important building block in the anaerobic lifestyle of *Prevotella* spp.

Other, closely related members of gastrointestinal microbiomes such as *Bacteroides fragilis* and *B. thetaiotaomicron* might also benefit from NADH:fumarate oxidoreduction performed by the NQR together with the fumarate reductase [60]. The fumarate reductase catalyzes the reaction of fumarate to succinate which is rapidly converted to propionate by *Prevotella* spp. [61]. However, part of the succinate formed is also excreted [15]. NADH:fumarate oxidoreduction under secretion of succinate could be a general strategy in members of the rumen microbiota to remove reducing equivalents and achieve redox balance in anaerobic metabolism. In the rumen, overall redox balance largely depends on methanogens which oxidize H^2^ produced by fermenting bacteria, thus representing the major hydrogen sink in this ecosystem. Once could speculate that when methanogenesis is inhibited, bacteria which are able to increase succinate production and secretion by increased NADH:fumarate oxidoreduction activity have an advantage. Indeed, it was reported that *Prevotella* spp. and *Clostridium aminophilum* increase in abundance when methanogenesis is inhibited [62]. Further studies are required to substantiate these assumptions.

## Figures and Tables

**Table 1:** Subunits of the Na^+^-translocating NADH:quinone reductase (NQR) and other respiratory enzymes produced by members of the Prevotellaceae family as identified by a metaproteomic survey of the rumen [17]. The entirety of identified proteins is listed in Table S1. Proteins belonging to the succinate dehydrogenase (succ.dehyd.) or fumarate reductase (fum. reduc.) were not identified unambiguously in all cases.

Uniprot Accession no.	Identified Proteins Species	Score (Max Quant)	Sequence Coverage (%)	Unique Peptides	Total Peptides
D5ESF6	NqrF	40.28	32.00	4	9
	*Prevotella ruminicola*				
D5ESG1	NqrA	11.67	19.60	5	8
	*Prevotella ruminicola*				
D5ESF9	NqrC	5.05	12.20	3	3
	*Prevotella ruminicola*				
A0A0D0J042	NqrF	3.99	11.30	2	3
	*Prevotella sp. P5-119*				
R5P524	NqrA	2.00	8.30	1	3
	*Prevotella sp. CAG:1092*				
D5ESG0	NqrB	1.67	2.60	1	1
	*Prevotella ruminicola*				
D1VWD5	NqrA	-2.00	7.00	2	3
	*Prevotella timonensis*				
A0A099BUQ4	NqrA	-2.00	6.00	1	3
	*Prevotella* sp. S7 MS 2				
D5EZ70	El. transp. complex RnfG	3.98	16.60	2	2
	*Prevotella ruminicola*				
D5EWF4	Succ. dehyd./fum. reduc., flavopr.	251.44	41.3	9	25
	*Prevotella ruminicola*				
D5EWF3	Succ. dehyd./fum. reduc., Fe-S pr.	133.55	51.6	1	13
	*Prevotella ruminicola*				
D3HYQ8	Succ. dehyd./fum. reduc. Fe-S sub.	9.28	53.6	0	13
	*Prevotella buccae*				
R6CJ62	Fumarate reductase, Fe-S pr.	9.13	47.2	0	11
	*Prevotella copri*				
A0A096C9D9	Succinate dehydrogenase	7.36	22.7	1	15
	*Prevotella amnii*				
D8DXM5	Fumarate reductase, Fe-S pr.	5.58	45.2	0	11
	*Prevotella bryantii* B_1_4				
L0JDX3	Menaqui.-Succ. dehyd./fum. reduc.	3.93	17.6	1	11
	*Prevotella dentalis*				
L1MZ30	Succ. dehyd./fum. reduc., flavopr.	3.78	26.4	1	16
	*Prevotella saccharolytica*				
R7F4R5	Succ. dehyd./fum. reduc., flavopr.	2.67	19	0	12
	*Prevotella* sp. CAG:485				
D7NDJ3	Fumarate reductase, Fe-S pr.	2.50	40.5	2	10
	*Prevotella oris*				
U2QH41	Succ. dehyd./fum. reduc., flavopr.	1.45	17.9	1	12
	*Prevotella baroniae*				
A0A096BN50	Succinate dehydrogenase	1.18	46.4	0	12
	*Prevotella buccalis*				
F8NCB8	Succinate dehydrogenase sub. B	-2.00	17.5	0	4
	*Prevotella multisaccharivorax*				
R5GTK6	Succ. dehyd./fum. reduc. Fe-S sub.	-2.00	44	0	10
	*Prevotella* sp. CAG:755				

**Table 2:** Subunits of respiratory, NADH-converting complexes and other respiratory enzymes identified in the two proteome fractions (membrane proteins, MP and cell debris, CD) of *P. bryantii* B_1_4. The entirety of identified proteins is listed in Table S2.

SampleFraction	Uniprot Accession no.	Identified Proteins of *Prevotella bryantii* B_1_4	Score (Mascot/PD)	Sequence Coverage (%)	Unique Peptides	Total Peptides
CD	D8DWC1	NqrA	275.00	17.37	7	7
	D8DWB9	NqrC	151.17	30.95	6	6
	D8DYE1	El. transp. complex RnfG (FMN)	95.90	23.66	3	3
	D8DWN9	NuoCD	117.97	7.82	3	3
	D8DWN7	NuoI	63.74	7.25	2	2
	D8DWP0	NuoB	57.69	9.45	2	2
	D8DXM5	Fumarate reductase, Fe-S pr.	619.21	51.19	13	13
	D8DXM6	Succinate dehydrogenase	475.15	22	15	15
MP	D8DWB9	NqrC	137.82	17.14	3	3
	D8DWC1	NqrA	113.57	11.58	5	5
	D8DYE1	El. transp. complex RnfG (FMN)	196.68	32.82	5	5
	D8DXV4	El. transp. complex RnfG	110.26	15.26	2	2
	D8DWN9	NuoCD	36.65	3.82	2	2
	D8DXM5	Fumarate reductase, Fe-S pr.	477.08	51.19	14	14
	D8DXM6	Succinate dehydrogenase	243.90	15.48	11	11

**Table 3:** Sequence identities inferred from multiple sequence alignments of the NQR subunits with the corresponding subunits of the RNF complex and the most similar subunits of the NDH-I (Nuo). Alignments were performed with Clustal Omega (European Bioinformatics Institute, ebi.ac.uk/Tools/msa/clustalo/).

NQR Subunit	Uniprot Accession No.	RNF Subunit	Uniprot Accession No.	Identity (%)	NDH-I Subunit	Uniprot Accession No.	Identity (%)
NqrA	D8DWC1	RnfC	D8DXV6	25.22	NuoH	D8DWN8	18.44
NqrB	D8DWC0	RnfD	D8DXV5	30.28	NuoK	D8DWN5	26.88
NqrC	D8DWB9	RnfG	D8DYE1	27.05	NuoI	D8DWN7	26.83
NqrD	D8DWB8	RnfE	D8DXV3	37.36	NuoN	D8DX02	19.08
NqrE	D8DWB7	RnfA	D8DXV2	44.50	NuoL	A0A1H9A8K0	16.67
NqrF	D8DWB6	RnfB	D8DXV7	17.34	NuoCD	D8DWN9	17.48

**Table 4:** Subunits of the NQR, RNF, NDH-I (Nuo), and other respiratory enzymes identified from membranes solubilized with 1% or 2% (*w*/*v*) Triton X-100. Bands were excised from the BN-PAGE as indicated in Figure S1. The entirety of identified proteins is listed in Table S3.

Protein Band	Uniprot Accession No.	Identified Proteins of *Prevotella bryantii* B_1_4	Score (Mascot/PD)	Sequence Coverage (%)	Unique Peptides	Total Peptides
Triton 1%—A	D8DWC1	NqrA	259.37	24.50	10	10
	D8DXV4	El. transp. complex RnfG	87.48	16.84	2	2
	D8DWN8	NuoH	98.71	5.77	2	2
	D8DXM6	Succinate dehydrogenase	1843.34	41.88	32	32
	D8DXM5	Fumarate reductase, Fe-S pr.	1272.00	63.89	16	16
Triton 1%—B	D8DWC1	NqrA	1126.93	60.58	26	27
	D8DWB6	NqrF	386.31	18.01	7	7
	D8DWB9	NqrC	272.89	40.48	7	8
	D8DWC0	NqrB	55.66	11.43	3	3
	D8DWB8	NqrD	41.65	4.78	2	2
	D8DWN8	NuoH	115.46	9.62	4	4
	D8DWN7	NuoI	90.35	16.58	3	3
	D8DX02	NuoN	81.47	4.50	2	2
	D8DWP0	NuoB	70.53	6.30	2	2
	D8DXM6	Succinate dehydrogenase	814.30	29.59	22	22
	D8DXM5	Fumarate reductase, Fe-S pr.	605.93	48.02	12	12
Triton 1%—C	D8DWC1	NqrA	1233.90	69.04	33	33
	D8DWB9	NqrC	348.79	41.43	8	9
	D8DWB6	NqrF	160.70	10.19	4	4
	D8DWC0	NqrB	44.90	6.49	2	2
	D8DXV4	El. transp. complex RnfG	88.64	15.26	2	2
	D8DWN8	NuoH	133.20	9.89	5	5
	A0A1H9A8K0	NuoL	110.73	5.13	3	3
	D8DWP0	NuoB	66.30	6.3	2	2
	D8DWN9	NuoCD	61.14	4.2	2	2
	D8DWN7	NuoI	39.70	11.4	2	2
	D8DXM6	Succinate dehydrogenase	120.15	6.22	5	5
	D8DXM5	Fumarate reductase, Fe-S pr.	99.83	9.92	2	2
Triton 2%—A	D8DWC1	NqrA	103.85	11.58	4	4
	D8DWN8	NuoH	73.76	5.77	2	2
	D8DXM6	Succinate dehydrogenase	2189.86	41.88	32	32
	D8DXM5	Fumarate reductase, Fe-S pr.	1159.13	60.71	15	15
Triton 2%—B	D8DWC1	NqrA	791.13	45.66	19	20
	D8DWB6	NqrF	255.86	14.45	6	6
	D8DWB9	NqrC	176.80	18.57	4	4
	D8DWN8	NuoH	122.62	9.62	4	4
	D8DWP0	NuoB	67.36	6.30	2	2
	D8DWN9	NuoCD	63.92	4.20	2	2
	D8DXM6	Succinate dehydrogenase	822.69	26.25	18	18
	D8DXM5	Fumarate reductase, Fe-S pr.	537.00	54.37	13	13
Triton 2%—C	D8DWC1	NqrA	1218.80	63.25	30	30
	D8DWB6	NqrF	464.91	24.64	8	9
	D8DWB9	NqrC	242.60	34.76	6	7
	D8DWC0	NqrB	66.45	11.17	3	3
	D8DWB8	NqrD	41.62	4.78	2	2
	D8DXV4	El. transp. complex RnfG	25.98	11.58	2	2
	D8DWN8	NuoH	118.11	7.69	4	4
	D8DWN7	NuoI	104.26	16.58	3	3
	D8DWP0	NuoB	78.14	8.66	3	3

## Supplementary Materials

The following are available online at https://www.mdpi.com/2076-2607/7/5/117/s1, Figure S1: BN-PAGE of *P. bryantii* B_1_4 membranes solubilized with 1% or 2% (w/v) Triton X-100. The protein bands that were excised for subsequent LC–MS/MS analysis are indicated, Table S1: Prevotellaceae-derived proteins identified in the rumen ecosystem. Quantification parameters of MaxQuant (v. 1.5.3.8) and the prediction parameters of CELLO (v. 2.5), TMHMM Server (v. 2.0) and PRED-TMBB2 for subcellular localization, α-helices and ß-barrel outer membrane proteins, Table S2: Entirety of proteins identified in the cell debris (CD) and membrane protein (MP) fractions of *P. bryantii* B_1_4. Identification parameters of the Thermo Proteome Discoverer software (v. 1.4.1.14) and the Mascot engine (v. 2.4) and the prediction parameters of CELLO (v. 2.5), TMHMM Server (v. 2.0) and PRED-TMBB2 for subcellular localization, α-helices and ß-barrel outer membrane proteins, Table S3: Entirety of proteins detected in bands A, B, and C from the BN-PAGE containing isolated membranes of *P. bryantii* B_1_4 that were solubilized using 1% and 2% Triton X-100. Identification parameters of the Thermo Proteome Discoverer software (v. 1.4.1.14) and the Mascot engine (v. 2.4).
