# Supplementary figures and images for: Occurrence and Function of the Na+-Translocating NADH:Quinone Oxidoreductase in Prevotella spp
Source: Microorganisms. 2019 Apr 27;7(5):117. doi: 10.3390/microorganisms7050117 (PMC6560451; doi:10.3390/microorganisms7050117)

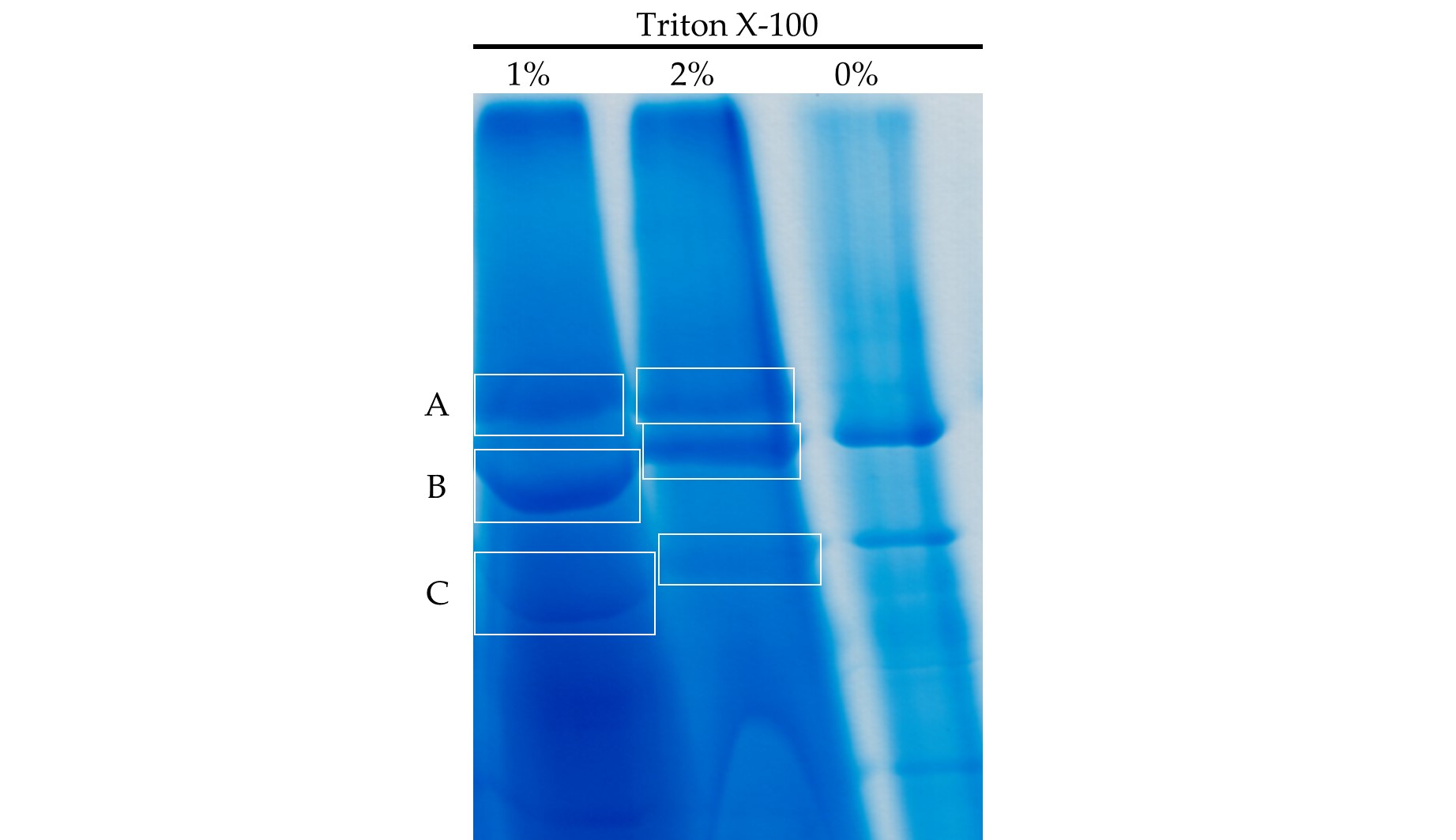

Supplement: Supplementary file 1 [file microorganisms-07-00117-s001.zip › Supplementary material/Figure S1.jpg]
